# Knockdown of RhoC Inhibits Oral Squamous Cell Carcinoma Cell Invasion and Metastasis via Regulation of HMGA2

**DOI:** 10.1155/2021/6644077

**Published:** 2021-01-12

**Authors:** Feng Gao, Panpan Yin, Yanlin Wu, Jinlin Wen, Ying Su, Xinyan Zhang

**Affiliations:** Beijing Institute of Dental Research, Beijing Stomatological Hospital and School of Stomatology, Capital Medical University, Beijing, China

## Abstract

Ras homolog family member C (RhoC) is an important component of intracellular signal transduction and its overexpression has been reported to be involved in regulating tumor proliferation, invasion, and metastasis in various malignant tumors. However, its role and underlying mechanism in oral squamous cell carcinoma (OSCC) still remain obscure. In our study, RhoC expression, its relation with clinical stages, and survival rate in OSCC were analyzed using datasets from The Cancer Genome Atlas (TCGA). Next, a RhoC knockdown cell model was established in vitro, and the effects of RhoC knockdown in OSCC cells were detected by the MTT assay, colony formation assay, transwell invasion assay, scratch assay, and F-actin phalloidin staining. An in vivo tongue-xenografted nude mouse model was established to measure the effects of knockdown of RhoC on tumor cell growth and lymph node metastasis. A mechanism study was conducted by real-time PCR and immunocytochemistry. The results of TCGA analysis showed that RhoC was overexpressed in OSCC tumor tissues. In vitro assays indicated that knockdown of RhoC did not have much effect on OSCC cell growth but significantly suppressed cell colony formation, invasion, and migration abilities, and F-actin polymerization was also reduced. The tongue-xenografted in vivo model demonstrated that knockdown of RhoC suppressed OSCC cell growth and inhibited metastasis to the superficial cervical lymph nodes. Further mechanism studies showed that knockdown of RhoC downregulated HMGA2 expression, and HMGA2 expression was highly correlated with RhoC expression in OSCC tumor tissues via the analysis of TCGA datasets. Overall, our study showed that knockdown of RhoC inhibited OSCC cells invasion and migration in vitro and OSCC cell growth and lymph node metastasis in vivo. Moreover, the potential mechanisms involved in these activities may be related to the regulation of HMGA2 expression. The RhoC gene could serve as a promising therapeutic target for OSCCs in the future.

## 1. Introduction

Oral squamous cell carcinoma (OSCC) is the tenth leading cause of cancer-related deaths worldwide, reaching 145,400 deaths in 2012 [1]. Despite the advances in treatment strategies, the five-year survival rate for OSCC has been less than 50% for the last three decades [2]. OSCC causes cervical lymph node metastasis due to the abundance of lymphatic vessels in the oral area [3]. The high OSCC mortality is considered strongly associated with the local invasive properties of tumor cells and with lymph node metastasis. Therefore, identifying the mechanisms underlying the invasion and metastatic properties of OSCC is urgently needed to improve patient outcomes.

Studies have shown that the abnormal activation of the Rho family of GTPases, components of the Ras homology protein family, plays a crucial role in a wide range of cell activities including cell proliferation, differentiation, apoptosis, cell adhesion, and invasive and metastatic potential of tumor cells [4, 5]. As an important member of the Rho GTPase family member, RhoC plays a significant role in the invasion and metastasis of malignant tumors by influencing epithelial-mesenchymal transition (EMT), extracellular matrix degradation, cell migration, and tumor angiogenesis [6]. RhoC is increasingly reported to be involved in the malignant potential of tumors, such as breast cancer [7], lung cancer [8], gastric cancer [9], colon cancer [10], prostate cancer [11], and head and neck squamous cell carcinoma (HNSCC) [12]. In particular, RhoC overexpression is associated with the metastatic behavior of HNSCC, whereas reduced RhoC expression significantly weakens tumor mobility and invasion [13]. Specifically, RhoC expression has been associated with tumor-node-metastasis frequently observed in OSCC [14]. Conversely, in a prostate cancer study, RhoC expression did not contribute to cell motility but only promoted cell invasion [15]. Furthermore, it has been reported that there is no correlation between the expression of RhoC levels and the histopathological grading of OSCC in situ. Despite this progress in the understanding of the involvement of RhoC in invasion and metastasis of tumors, further investigations in vivo and in vitro are urgently needed to explore the role of RhoC in OSCC and its effects on downstream signaling molecules to provide scientific validation as a clinical target for cancer treatment.

In this study, we analyzed datasets retrieved from the TCGA to explore the correlations between RhoC expression and clinicopathological features of OSCC. Further, we established a RhoC knockdown OSCC cell line model to explore the biological performance and to define the potential functions and mechanisms of RhoC-mediated activity in OSCC not only in vitro, but also in vivo. Our results indicated that the overexpression of RhoC was closely related to tumor metastasis of OSCC, while knockdown of RhoC restrained the invasion and metastasis capability of OSCC cells in vivo and in vitro, and these effects may be related to the regulation of HMGA2 expression. Further, the RhoC gene may serve as a potential therapeutic target for OSCC in the future.

## 2. Materials and Methods

### 2.1. Cancer Data Collection and Preprocessing

The oral cancer data including the gene data, isoform RSEM data, and clinical data were systematically searched and downloaded from the UCSC Xena browser (GDC hub: https://gdc.xenahubs.net). The following search parameters were used: oral cavity, oral tongue, buccal mucosa, lip, alveolar ridge, hard palate, floor of mouth. Then, the R software version 3.6.1 was used to transform and analyze RNA-sequencing data (FPKM values) [16]. Kaplan–Meier analysis with the log-rank test was used to analyze the overall survival.

### 2.2. In Vitro Culture of OSCC Cell Lines

The OSCC cell lines CAL-27 and SCC-15 were obtained from the Beijing Stomatological Hospital Research Institution, Capital Medical University. The cells were routinely cultured in DMEM high glucose or DMEM:F12 medium (Invitrogen Life Science, Carlsbad, CA) supplemented with 10% fetal bovine serum (FBS), 100 *μ*g/mL streptomycin, and 100 U/mL penicillin, and incubated at 37°C in a humidified 5% CO_2_ incubator.

### 2.3. Construction of RhoC/shRNA Plasmid and Transfection

The vector LVRU6MP coding for RhoC/shRNA and the control vector encoded with mock shRNA were constructed by GeneCopoeia, Inc. (Rockville, MD). The 293Ta lentiviral packaging cells were used to package lentiviral particles and to generate recombinant lentiviral particles using the Lenti-Pac HIV Expression Packaging Kit and psi-LVRU6MP/RhoC/shRNA. Lentiviruses containing the RhoC/shRNA gene were transfected into CAL-27 (6 × 10^4^) and SCC-15 (1.5 × 10^5^) cells in 6-well dishes with Polybrene (GenePharma, China). A lentiviral transfer vector that expressed the mCherry protein was used as control. The transfected cells were selected by puromycin (Sigma-Aldrich, MO) treatment and expanded for further experiments. The viability of transfected cells remained stable.

### 2.4. Real-Time Polymerase Chain Reaction (RT-PCR)

Total RNA was extracted from transfected cells using Trizol reagent (ComWin Biotech Co., Ltd., China) according to the manufacturer's protocol, and reverse transcription was performed using the Super Reverse Transcription cDNA Synthesis Kit (ComWin Biotech). PCR was performed with the ULtraSYBR Mixture (Low ROX) (ComWin Biotech). The primer sets for human RhoC (Catalog number: QRP20382) were provided by GeneCopoeia Inc. The primer sets for GAPDH (forward, 5′-CATGGGTGTGAACCATGAGAAGTAT-3′ reverse, 5′-GACTGTGGTCATGAGTCCTTCCA-3′) and HMGA2 (forward, 5′-GCCAAGAGGCAGACCTAGGAAA-3'; reverse, 5′-CATGGCAATACAGAATAAGTGGTCA-3′) were provided by Sangon Biotech Co., Ltd. (China). The ∆∆CT method was applied for quantification analysis [17], and GAPDH was used as an endogenous control.

### 2.5. Immunocytochemistry

The expression of RhoC and HMGA2 was detected by immunocytochemistry (IHC) at the protein level. The control and shRNA transfected CAL-27 (1 × 10^5^) and SCC-15 (2 × 10^4^) cells were, respectively, cultured in 24-well plates overnight. Next, 10% neutral buffer formalin fixative was used to fix cells for 30 min and cells were incubated with Triton X-100 for 10 min. Goat serum (10%) was used to block the cells for 1 h, which were subsequently washed with PBS 3 times. Next, cells were incubated with 3% goat serum containing antibody RhoC (ab180785, Abcam, 1 : 400) and HMGA2 (ab97276, Abcam, 1 : 500), respectively, overnight at 4°C. Cells were washed with PBS and were incubated with 3% goat serum containing the second antibody for 1 h and then stained with a diaminobenzidine (DAB) kit (ComWin Biotech Co., Ltd., China). The cells were then photographed under an inverted microscope.

### 2.6. MTT Assay

To evaluate cell proliferation, the transfected cells were plated in 96-well plates. Following culturing for 24, 48, and 72 h at 37°C, 5% CO_2_, a 20 *μ*L volume of MTT (Sigma, USA) solution (5 mg/mL) was added to each well and cultured for an additional 4 h. Next, 200 *μ*L dimethyl sulfoxide (DMSO, Sigma, USA) was added to each well to dissolve the reaction products, and a microplate reader (Molecular Devices, Sunnyvale, CA) was used to measure the optical density (OD) value obtained at 490 nm wavelength.

### 2.7. Colony Formation Assay

To evaluate cell colony formation ability, the transfected cells (1000/plate) were seeded in 60 mm culture dishes. The cells were fixed with 10% neutral buffer formalin fixative and were stained with crystal violet solution (Beyotime, China) after 12 days of culture. The colonies were photographed and counted.

### 2.8. Transwell Invasion Assay

To evaluate cell invasion, transfected cells suspended in medium containing 2% bovine serum albumin (BSA, VWR, Radnor, PA) were seeded above the transwell membranes coated with Matrigel (BD Bioscience, Franklin Lakes, NJ) in the upper chamber (8 *μ*m pore size; Corning, Corning, NJ). Medium containing 10% FBS was added to the lower chamber. After a 48 h incubation at 37°C, 5% CO_2_, the remaining cells on the upper membrane were removed using a cotton swab, and the cells that had passed through the membrane were fixed with 10% neutral buffer formalin fixative and then stained with crystal violet solution. Under an inverted microscope, the membranes were photographed and the cell numbers were counted.

### 2.9. Scratch Migration Assay

To evaluate cell migration, transfected cells were inoculated into 6-well plates. After the cells were cultured for 24 h, a uniform wound in a straight line was drawn using a pipette tip in each well; cells were washed with PBS to remove detached cells. Next, the cells were cultured in medium containing 2% FBS at 37°C, 5% CO_2_. The wells were photographed and the closure or filling-in of the wounds was evaluated at 24, 48, 72, and 96 h under microscopy (OLYMPUS IX71, Tokyo, Japan) with 400× magnification.

### 2.10. Phalloidin Staining

To evaluate F-actin polymerization, the transfected cells were fixed in 40 g/L formaldehyde for 30 min after being inoculated into 24-well plates and cultured for 24 h. After permeabilization with 0.1% Triton X-100 for 10 min and blocking in 1% BSA for 30 min, the cells were incubated with 5 *μ*g/mL rhodamine-conjugated phalloidin (Sigma, USA) for 1 h. The cells were then counter-stained with DAPI. F-actin images were acquired under fluorescence and photographs were using a microscope (Olympus IX71, Japan) with 200x magnification. The mean optical density (MOD) value of F-actin polymerization was detected.

### 2.11. BALB/c Nude Mice Tongue-Xenografted Model

BALB/c nude mice (male, aged 6 weeks) were purchased from SPF Biotechnology Co. Ltd. (China). After adapting to the environment for a week, 35 BALB/c nude mice were randomly assigned into three groups and tagged: group A, blank control group; group B, inoculated CAL-27/RhoC/shRNA cells; and group C, inoculated CAL-27/control cells. The nude mice were inoculated with OSCC cells (25 *μ*L in PBS, 5 × 10^6^) at the lateral part of the mouse tongue. Mouse body weights were measured every 3 days and the mice were sacrificed after 12 days.

### 2.12. Hematoxylin and Eosin Staining

After the mice were sacrificed, tongue and lymph node samples of each mouse were taken and fixed in neutral formalin. Tissue samples were embedded in paraffin wax and sectioned. Tissue sections (5 *μ*m thick) were stained with hematoxylin and eosin (H&E) according to the manufacturer's protocol; images were taken and assessed using an optical microscope (OLYMPUS, BX61).

### 2.13. Statistical Analysis

Data were expressed as mean ± standard deviation (SD). SPSS Statistics v25.0 (IBM, Armonk, NY) was used to assess statistical significance using Student's *t*-test for paired comparisons and the chi-square test for sample rates. Statistical significance was set at *P* < 0.05.

## 3. Results

### 3.1. Assessment of RhoC in Oral Cancer Patients Analyzed in TCGA Dataset

**Figure 1:**
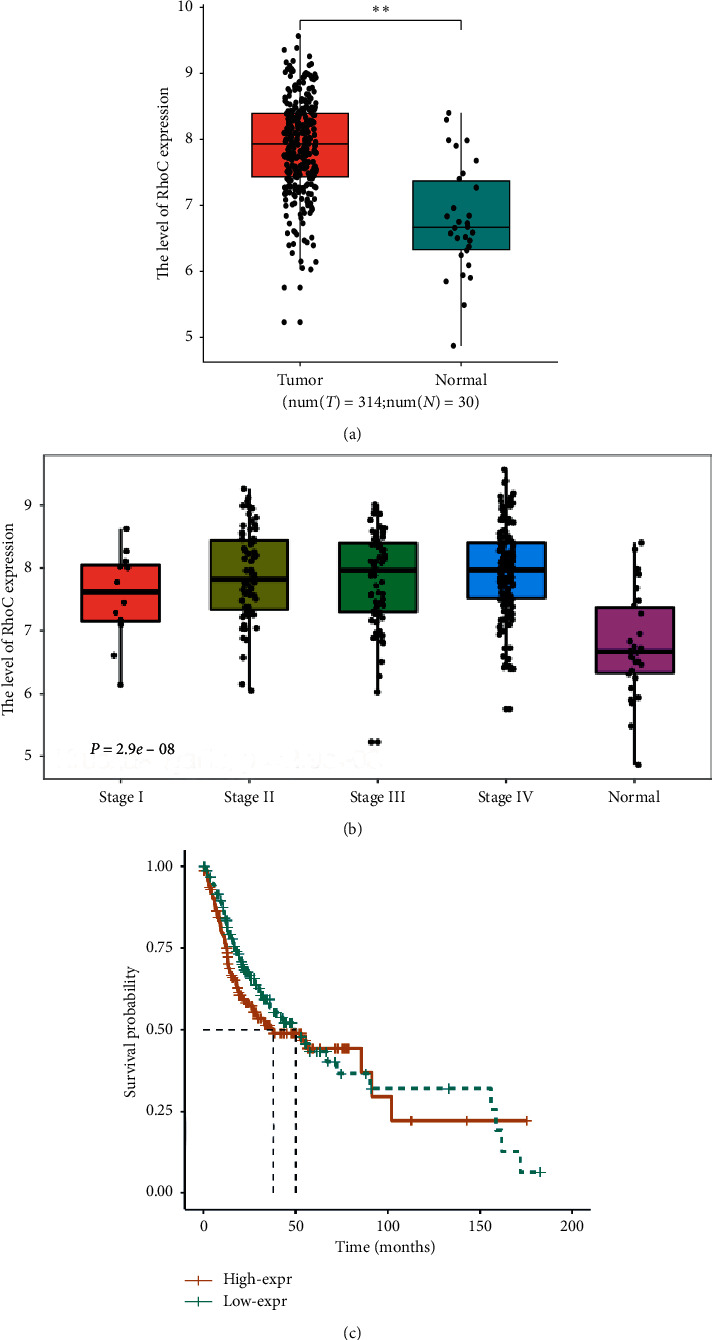
Assessment of RhoC in OSCC patients analyzed in TCGA datasets. (a) The expression levels of RhoC in OSCC tumor tissues are higher than in normal human epithelial tissues analyzed in the TCGA dataset (*P* < 0.01). (b) Relative RhoC expression levels at each stage, the expression levels of RhoC in stages II–IV are higher than in stage I. (c) The association of RhoC expression with overall survival (OS) in OSCC patients (*P*=0.281).

The expression of RhoC in oral cancer was analyzed in datasets from TCGA. RhoC expression was significantly higher in tumor tissues (*n* = 314) than in normal epithelial tissues (*n* = 30, Figure 1(a), *P* < 0.01). The level of RhoC expression was higher in stage II-IV patients than in stage I patients (Figure 1(b), *P* < 0.01). Overall survival (OS) analysis indicated that patients with high RhoC expression had poorer OS than cancer patients with lower RhoC expression, but the difference was not statistically significant (Figure 1(c), *P*=0.281). The above results indicated that RhoC was overexpressed in tumor tissues and the expression of RhoC correlated with tumor progression.

### 3.2. Establishment of Knockdown Model of RhoC in OSCC Cell Lines

**Figure 2:**
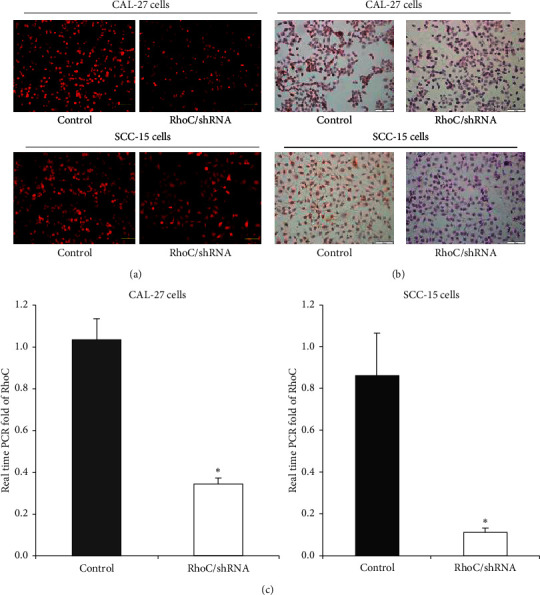
RhoC gene knockdown in OSCC cells. (a) Stable transfection of lentivirus with scrambled or RhoC/shRNA in OSCC cells is indicated by red fluorescence (magnification, ×200). (b) Expression of RhoC at the protein level is significantly decreased in RhoC/shRNA cells by ICC, bar = 100 *μ*m. (c) Expression of RhoC at the RNA level is significantly decreased in RhoC/shRNA cells compared to controls as assessed by RT-PCR. ^*∗*^*P* < 0.05 versus control.

To explore the biological role of RhoC in OSCC progression, an HIV-based lentiviral plasmid containing RhoC/shRNA was constructed and the lentiviruses were transfected into OSCC cell lines (CAL-27 and SCC-15) to knockdown RhoC expression (Figure 2). OSCC cells transfected with plasmids containing scrambled shRNA served as control. Stably transfected cells were selected by puromycin treatment and the efficiency of knockdown of RhoC was detected by RT-PCR and ICC. The RNA expression of RhoC in the RhoC/shRNA group was significantly lower than in the control group (Figure 2(c)). Similarly, the protein expression of RhoC in the RhoC/shRNA group was also significantly lower than controls (Figure 2(b)).

### 3.3. Knockdown of RhoC Had Minimal Effects on Cell Proliferation but Inhibited Colony Formation In Vitro

**Figure 3:**
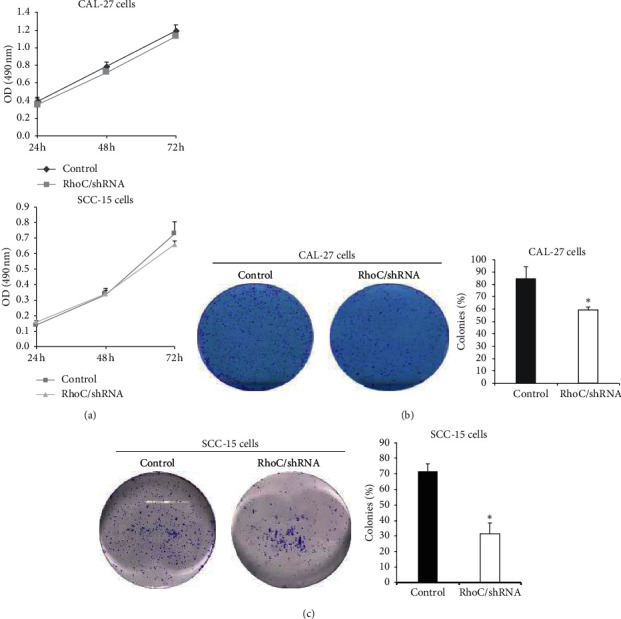
Knockdown of RhoC has no effect on OSCC cell proliferation but could inhibit colony formation. (a) Knockdown of RhoC has no effect on the growth of OSCC cells (CAL-27 and SCC-15 cells) as shown by the MTT assay. (b) Knockdown of RhoC inhibits the colony formation of CAL-27 compared with control group. ^*∗*^*P* < 0.05 versus control. (c) Knockdown of RhoC inhibits the colony formation of SCC-15 compared with control group versus control.

To further explore the role of RhoC in OSCC, an in vitro MTT assay investigating cell proliferation was performed. The results indicated that knockdown of RhoC did not exert any effect on the growth of either OSCC cell line (CAL-27 and SCC-15, Figure 3(a)). However, the colony formation assay revealed that RhoC knockdown markedly reduced the number of colonies formed in both OSCC cell lines (Figures 3(b) and 3(c)).

### 3.4. Knockdown of RhoC Suppressed Invasion, Migration, and F-Actin Polymerization

**Figure 4:**
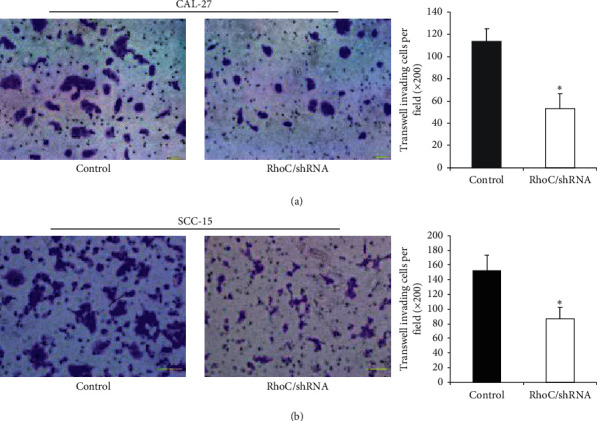
Knockdown of RhoC decreases the invasive ability of OSCC cells ((a) CAL-27 and (b) SCC-15 cells) in the transwell invasion assay (magnification, ×200). ^*∗*^*P* < 0.05 versus control. Bar = 100 *μ*m.

**Figure 5:**
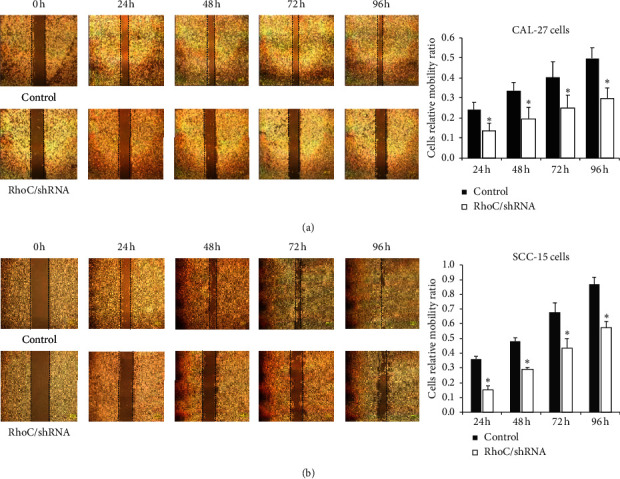
Knockdown of RhoC suppresses the migration ability of OSCC cells ((a) CAL-27 and (b) SCC-15 cells) in the scratch migration assay. ^*∗*^*P* < 0.05 versus control. Bar = 500 *μ*m.

**Figure 6:**
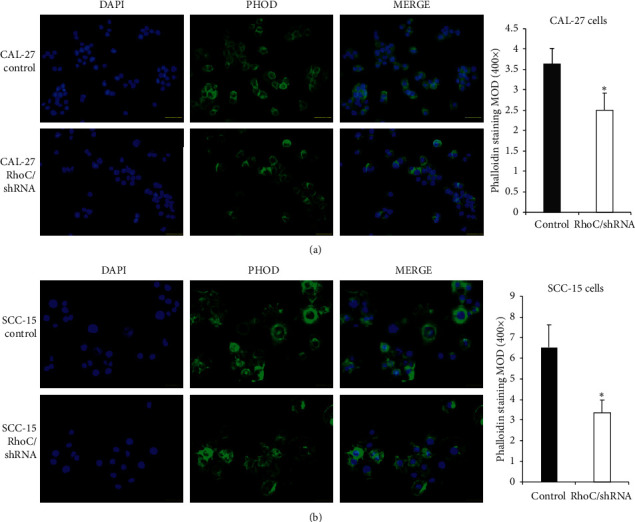
Knockdown of RhoC suppresses the F-actin polymerization of OSCC cells ((a) CAL-27 and (b) SCC-15 cells). ^*∗*^*P* < 0.05 versus control. Bar = 50 *μ*m.

The invasion assay indicated that knockdown of RhoC markedly reduced the number of invading CAL-27 and SCC-15 OSCC cell lines (Figure 4). The scratch migration assay also indicated that RhoC knockdown slowed down the relative migration ratio of CAL-27 cells in a time-dependent manner, and similar results were obtained using SCC-15 cells (Figure 5). Furthermore, phalloidin labeling of F-actin was also significantly lower in the RhoC/shRNA group than in the control group as indicated by the MOD value (Figure 6); the cells appeared smaller and less able to spread out in the RhoC/shRNA group OSCC cell lines. These data indicated that the downregulation of RhoC suppressed the invasion, migration, and cell mobility of OSCC cells in vitro.

### 3.5. Knockdown of RhoC Inhibited CAL-27 Cell Growth in Tongue Xenografts and Suppressed Metastases to the Superficial Cervical Lymph Nodes in Nude Mice

**Figure 7:**
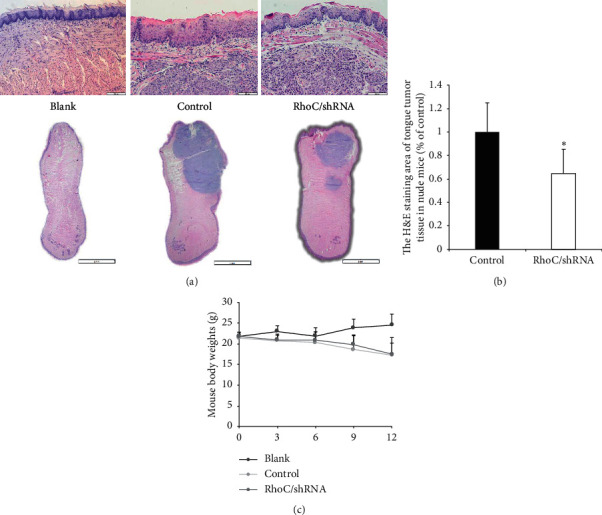
Knockdown of RhoC suppresses CAL-27 cell growth in the tongue-xenografted nude mice model. (a) H&E staining of the tongue in nude mice, bar above = 200 *μ*m, bar below = 2 mm. The degree of epithelial cell proliferation was lower in the RhoC/shRNA group than in controls. (b) The proportion of tumor tissue in the tongue of nude mice is lower in RhoC/shRNA group than in controls. ^*∗*^*P* < 0.05. (c) Comparison of mouse body weights.

**Figure 8:**
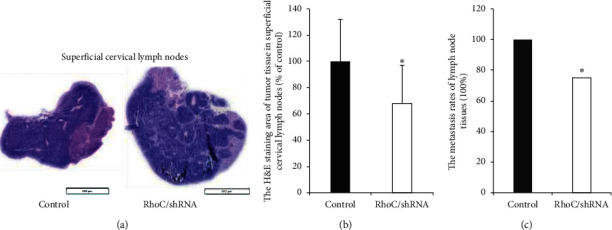
Knockdown of RhoC inhibits CAL-27 cell metastasis to the superficial cervical lymph nodes and reduces the metastasis rate of lymph nodes. (a) H&E staining of the tumor mass in the superficial cervical lymph nodes, bar = 500 *μ*m. (b) The proportion of metastatic tumor tissue in the superficial cervical lymph nodes (% of control) is lower in the RhoC/shRNA group than in controls. ^*∗*^*P* < 0.05 versus control. (c) The metastasis rate of lymph node tissues is lower in the RhoC/shRNA group than in controls. ^*∗*^*P* < 0.05 versus controls.

OSCC mostly occurs in the tongue and is prone to lymph node metastasis. Thus, a nude mouse tongue-xenografted model was established to investigate the effects of decreased RhoC expression in vivo by injecting CAL-27/RhoC/shRNA cells and control cells into the tongues of nude mice. Mice were observed for 12 days. Subsequently, H&E staining was used to determine the proportion of tumor area in excised mouse tongue and in lymph nodes tissues, and the metastasis rate of lymph nodes was determined. The H&E staining of tongue showed that compared to control mice the injection of tumor cells could lead to epithelial hyperplasia: the degree of epithelial cell proliferation was significantly lower in the RhoC/shRNA group than in the control group (Figure 7(a)). We determined that the proportion of tumor area in tongues of the RhoC/shRNA group was significantly lower (64.7%) than in the control group (Figure 7(b)). Additionally, the metastatic tumor area was reduced in the superficial cervical lymph nodes in the RhoC/shRNA group (Figure 8(a)). The proportion of metastatic tumor cells in the superficial cervical lymph nodes was reduced by 32% compared to control mice (Figure 8(b)), and the metastatic rate was significantly reduced from 98.5% (control group) to 68.0% (RhoC/shRNA group) (Figure 8(c)). Overall, the knockdown of RhoC expression gave rise to a significant reduction in the tumor area in the xenografted tongue and lymph node tissues, and similarly the relative lymph node metastatic rate was also reduced to lower than that of the control group. Above all, our results provided evidence that decreased RhoC expression reduced the invasion and metastasis of OSCC cells in vivo.

### 3.6. Knockdown of RhoC Regulated HMGA2 Expression

**Figure 9:**
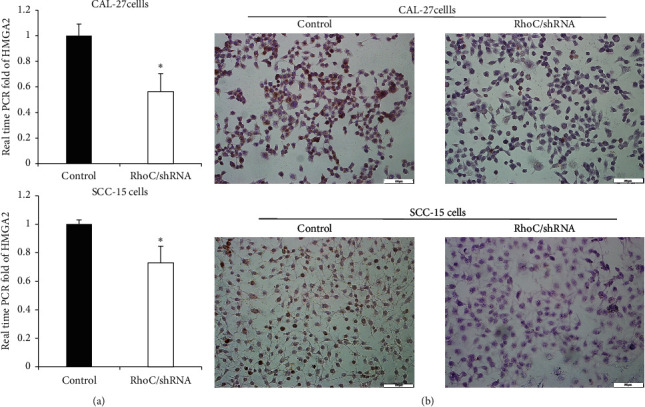
Knockdown of RhoC regulates the expression of HMGA2. (a) Relative RNA expression level of HMGA2 is lower in the RhoC/shRNA group. ^*∗*^*P* < 0.05 versus controls. (b) Protein expression of HMGA2 is lower in the RhoC/shRNA group by ICC, bar = 100 *μ*m.

**Figure 10:**
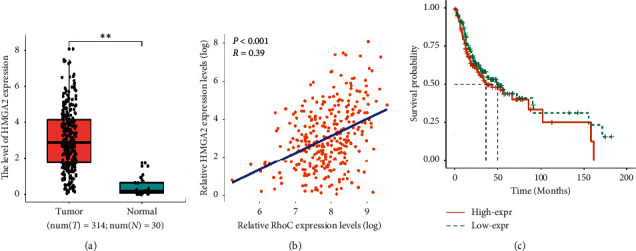
The analysis of HMGA2 in the TCGA datasets. (a) The expression of HMGA2 is higher in OSCC than in normal epithelial tissues analyzed in TCGA datasets. (b) RhoC is co-expressed with HMGA2 in the correlation analysis (Pearson's correlation) in OSCC tissues, *P* < 0.001, *R* = 0.39. (c) The association of HMGA2 expression with overall survival (OS) in OSCC patients *P*=0.267.

To further explore the mechanism of RhoC in OSCC cells, the expression of HMGA2 was examined in transfected OSCC cells. Both RNA and protein expression levels of HMGA2 were significantly reduced in the RhoC/shRNA group (Figure 9). In addition, we found there was a clinical significance supporting the RhoC-HMGA2 interaction using bioinformatics analysis. HMGA2 was overexpressed in OSCC tumor tissues (Figure 10(a)) and further there was a close correlation between the expressions of RhoC and HMGA2 (Figure 10(b), *P* < 0.01, *R* = 0.39). Survival analysis showed that high expression of HMGA2 was also associated with poor OS, but this association was not statistically significant (Figure 10(c), *P*=0.267, although it was statistically significant in HNSC, data not shown). These data indicated that the expression of HMGA2 was regulated by RhoC.

Overall, our study suggests that knockdown of RhoC expression is able to suppress the malignant biological behavior of OSCC, specifically the malignant properties of cell invasion, migration, tumor growth, and lymph node metastasis, and these might be achieved by regulating HMGA2 expression.

## 4. Discussion

Metastasis is an important hallmark of malignancy and a common poor prognostic factor for cancer patients [18]. As one of the most common cancers in humans, OSCC is prone to lymph node metastasis even at early stages. Many efforts have been attempted to identify molecular markers that could help predict cancer prognosis. The analysis of TCGA datasets showed that RhoC was overexpressed in tumor tissues and was associated with metastasis in OSCC patients, and these results were consistent with those of a clinical IHC study [19]. In previous studies, RhoC has been reported to participate in the regulation of cytoskeleton reorganization, and it influenced cell adhesion and migration [6]. In this study, we obtained similar results, whereby knockdown of RhoC effectively inhibited OSCC cell invasion and metastasis in vitro and in OSCC-xenografted nude mice. This evidence supports the role of RhoC in OSCC cells as an oncogene and is consistent with previous studies in a variety of cancer types and is supported by relevant clinical studies [11, 13, 20].

Although accumulating evidence has suggested that RhoC is associated with cell invasion and migration and it plays an important role in advanced tumors, evidence regarding the role of RhoC in regulating tumor cell proliferation has been controversial. In previous studies, knockdown of RhoC expression in a hepatoma cell line could significantly increase the percentage of interphase cells and thus inhibit cell proliferation [21]. However, in this study, we provide evidence indicating that the cell growth of OSCC cells in the RhoC knockdown group was not significantly inhibited compared to the cell growth in the control group in MTT proliferation assays. This suggested that downregulation of RhoC might not decrease OSCC cell proliferation, which is also consistent with studies using mouse models of lung cancer [22].

To date, the molecular mechanisms involved in RhoC promotion of tumor progression have not been fully understood. Previous studies have shown that RhoC promotes cancer development by regulating the expression of MMP genes in EMT [11, 21, 23]. However, changes in the levels of EMT markers such as E-cadherin and *β*-catenin were not observed in our study (data not shown), which suggests that the effects of RhoC on invasion and metastasis in OSCC cells may not be achieved by regulating EMT.

In this study, HMGA2 expression was regulated in OSCC cells following RhoC knockdown. Meanwhile, other studies have reported that HMGA2 was overexpressed in melanoma cancer [24], ovarian cancer [25], and nasopharyngeal cancer [26]. HMGA2 is considered to play a key role in the regulation of in cancer cell proliferation, apoptosis, migration, and invasion [27]. Furthermore, a previous investigation also suggested that the expression of HMGA2 significantly correlated with the invasion and survival of glioma cells [28]. HMGA2 was also found to be expressed at the invasive front of oral carcinomas [29]. These findings along with our data suggest that RhoC might affect OSCC cell malignant behavior by regulating the HMGA2 pathway.

Our studies provide novel insights into understanding the mechanisms underlying the malignant potential of OSCC and provide a rationale for novel strategies and therapeutic targets in OSCC. Nonetheless, further studies are needed to investigate the mechanisms underlying the effects of RhoC/HMGA2 pathway in the progression of OSCC.

In conclusion, the present study revealed that RhoC is overexpressed in OSCC tissues, knockdown of RhoC suppressed cell invasion and metastasis of OSCC cells in vitro and in vivo, and these effects might be connected to the regulation of HMGA2 expression. Therefore, clinical diagnosis may benefit from RhoC assessment, and the RhoC/HMGA2 signaling pathway could serve as a potential therapeutic target for the treatment of OSCC.

## Data Availability

All the data included in this study are available upon request by contact with the corresponding author. The clinical data about the relations of RhoC and OSCC patients can be obtained at the TCGA website (https://portal.gdc.cancer.gov/).

## Abbreviations

OSCC: Oral squamous cell carcinoma
RhoC: Ras homolog family member C
TCGA: The Cancer Genome Atlas
EMT: Epithelial-mesenchymal transition
DAB: Diaminobenzidine
H&E: Hematoxylin and eosin
FBS: Fetal bovine serum
DMSO: Dimethyl sulfoxide
MOD: Mean optical density
BSA: Bovine serum albumin
PBS: Phosphate buffered saline.
